# Battle of Arbuscular Mycorrhizal Fungi Against Drought Stress: A Gateway to Sustainable Agriculture

**DOI:** 10.3390/jof12010020

**Published:** 2025-12-27

**Authors:** Asfa Batool, Shi-Sheng Li, Hong-Jin Dong, Ali Bahadur, Wei Tu, Yan Zhang, Yue Xiao, Si-Yu Feng, Mei Wang, Jian Zhang, Hong-Bin Sheng, Sen He, Zi-Yan Li, Heng-Rui Kang, Deng-Yao Lan, Xin-Yi He, Yun-Li Xiao

**Affiliations:** 1 Key Laboratories of Economic Forest Germplasm Improvement and Comprehensive Resources Utilization of Hubei Province, Collaborative Innovation Center for the Characteristic Resources Exploitation of Dabie Mountains, College of Biology and Agricultural Resources, Huanggang Normal University, Huanggang 438000, China; shishengli@hgnu.edu.cn (S.-S.L.); hongjind@hgnu.edu.cn (H.-J.D.); tuwei@hgnu.edu.cn (W.T.); 13597691690@163.com (Y.Z.); 19027208617@163.com (Y.X.); 18271090191@163.com (S.-Y.F.); cara_sucheng@163.com (M.W.); 19507239655@139.com (J.Z.); 13387575534@163.com (H.-B.S.); 19871108046@163.com (S.H.); 13545485058@163.com (Z.-Y.L.); 19947737572@163.com (H.-R.K.); 18671863875@163.com (D.-Y.L.); 18771814643@163.com (X.-Y.H.); 2 Cryosphere and Eco-Environment Research Station of Shule River Headwaters, Key Laboratory of Cryospheric Science and Frozen Soil Engineering, Northwest Institute of Eco-Environment and Resources, Chinese Academy of Sciences, Lanzhou 730000, China; alibahadur@nieer.ac.cn

**Keywords:** root hydraulics, aquaporins, antioxidant defense, soil aggregation, water-use efficiency

## Abstract

Around 85% of all land plants have symbiotic relationships with arbuscular mycorrhizal (AM) fungi, microscopic soil fungi that build extensive filamentous network in and around the roots. These links strongly influence plant development, water uptake, mineral nutrition, and defense against abiotic stresses. In this context, the use of AMF as a biological instrument to enhance plant drought resistance and phenotypic plasticity, through the formation of mutualistic associations, seems like a novel strategy for sustainable agriculture. This review synthesizes current understanding on the mechanisms through which AMF alleviates drought stress in agriculture. We focus on how AMF help maintain nutrient and water homeostasis by modulating phytohormones and signaling molecules, and by orchestrating associated biochemical and physiological responses. Particular emphasis is placed on aquaporins (AQPs) as key water-and stress-related channels whose expression and activity are modulated by AMF to maintain ion, nutrient, and water balance. AMF-mediated host AQP responses exhibit three unique patterns under stressful conditions: either no changes, downregulation to limit water loss, or upregulation to promote water and nutrient uptake. Nevertheless, little is known about cellular and molecular underpinnings of AMF effect on host AQPs. We also summarize evidence that AMF enhance antioxidant defenses, osmotic adjustment, soil structure, and water retention, thereby jointly improving plant drought tolerance. This review concludes by outlining the potential of AMF to support sustainable agriculture, offering critical research gaps, such as mechanistic studies on fungal AQPs, hormonal crosstalk, and field-scale performance, which propose future directions for deploying AMF in drought-prone agroecosystems.

## 1. Introduction

The term “mycorrhiza” was coined to describe the intimate connection between plant roots and fungi, following recognition by botanists and mycologists for the pivotal role being played due to these symbiotic relationships in terrestrial ecosystems [1]. Among different mycorrhizal types, arbuscular mycorrhiza forms symbiosis with over 85% of terrestrial plant species [2]. This ubiquity underscores the fundamental importance of arbuscular mycorrhizal symbiosis in plant-fungal interactions and nutrient cycling. Microscopic soil fungi known as arbuscular mycorrhizal fungi (AMF) form an extensive filamentous network both inside roots (intraradical mycelium and arbuscules) and in the surrounding soil (extraradical mycelium) [3]. These networks substantially enhance the uptake of several nutrients, including potassium, zinc, copper, nitrate, ammonium, and phosphorus, thereby reshaping the nutritional status of the host plant. The physiological and nutritional requirements of the plant and fungus interact through a complex feedback process that regulates carbon allocation, symbiotic development, and nutrient exchange [4,5].

Plants colonized by AMF generally show improved mineral nutrition and increased environmental adaptability. Experimental evidence strongly supports the role of AMF in enhancing tolerance to multiple abiotic stresses, including drought [6], heavy metal pollution [7], extreme temperature [8], salinity, and cold weather [9]. In addition, symbiotic plants often indicate reduce incidence or severity of certain root diseases, as AMF can suppress or counteract specific soil-borne pathogens, partly through competition while partly through induced host resistance [10,11]. Similarly, Auge et al. [12] found that plants respond to drought stress through interaction with AMF, and this AMF play its role in physiological, nutritional, and structural adjustments which help in alleviating water deficit. A recent study has shown that AMF mitigate drought stress by altering hormonal balance and improving plant water status through enhanced water absorption via extraradical hyphae, which facilitates root hydraulic conductivity [13]. There is also consistent evidence that AMF can increase antioxidant activity, promote osmotic adjustment [14,15,16], and enhance nutrient acquisition under drought [17,18]. Taken together, these responses contribute to better growth and resilience of mycorrhizal plants under environmental conditions that threaten their survival.

There are many major crops being cultivated for human and animal consumption that naturally form mycorrhizal associations, and there is considerable potential to exploit these interactions to improve agricultural systems. As highlighted by Bahadur et al. [19], this potential depends on the deliberate selection in compatible and efficient plant-fungus combinations and also on management practices that foster mycorrhizal functioning. Such strategies align with the broader concept of sustainable agriculture, in which crop productivity is pursued in parallel with soil health and ecosystem integrity [20]. By mobilizing AMF to improve nutrient use efficiency and stress resilience, it may be possible to reduce dependency on synthetic fertilizers and pesticides, thereby lowering environmental footprints [21]. This approach seeks to balance environmental quality with the profitability and stability of crop production. In practice, farmers can support more sustainable and resilient systems by adopting practices that enhance soil structure and biological activity, optimize resource use, and harness natural processes [22]. The overarching goal is to move away from highly input-intensive systems towards more economically and ecologically responsible alternatives that maintain or extend the productive lifespan of agricultural land [23].

Several studies have demonstrated the role of AMF in plant responses to drought stress at physiological and biochemical levels, providing mechanistic insights into AMF-mediated drought tolerance in plants, as well as broader syntheses that discuss drought as one component within wider AMF abiotic stress interactions [6,24,25]. Building on this foundation, the present review aims to provide a crop-centered, mechanistically integrated synthesis on how AMF affects different mechanisms in plants such as aquaporins, root hydraulics, hormonal crosstalk, and antioxidant systems, and then soil physical properties. By linking these mechanisms explicitly to drought resilience and considering their implications for management, this review seeks to offer an updated and more unified perspective that how AMF can be strategically connected to enhance crop drought performance under water-limited conditions in the context of environmentally friendly agriculture.

## 2. Drivers and Consequences of Drought Stress in Agriculture

**Figure 1:**
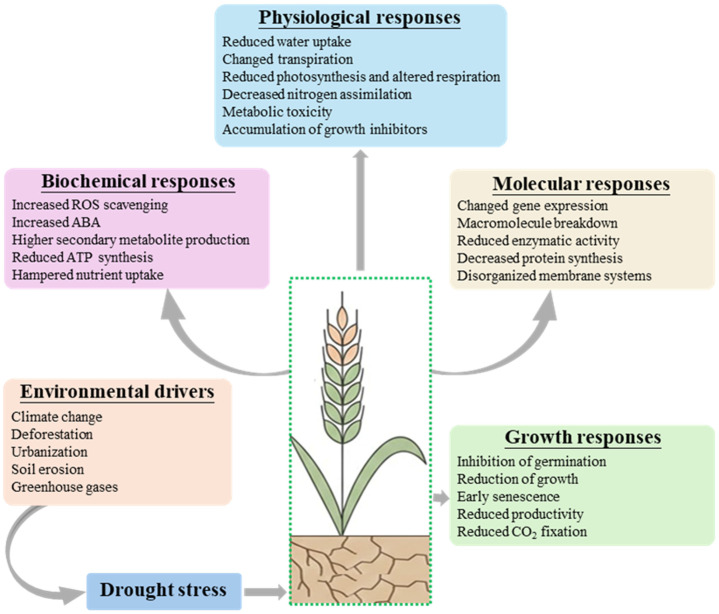
The morphological, physiological, and biochemical alterations that are essential for drought adaptation are illustrated graphically.

Global climate change, characterized by rising air temperature and increased atmospheric CO_2_ and CH_4_ levels, is the main driver for intensifying frequency and severity of drought events in agricultural ecosystems [26,27]. Altered rainfall distribution and more irregular precipitation patterns reduce effective rainfall for crops, while higher temperatures, dry winds, and prolonged heat episodes accelerate soil evaporation and evapotranspiration, thereby aggravating pre-existing drought deficits [28]. Beyond causing oil moisture depletion, global warming also alters plant internal water relations; higher temperatures increase vapor pressure deficit and promote greater transpirational water loss, further tightening the soil–plant–atmosphere water continuum (Figure 1) [29]. Projections suggest that if the global mean temperature rises by about 2 °C at the end of the century, up to one-fifth of the world’s population may be exposed to drought related impacts on water and food systems [30]. Although such projections are model-dependent and carry uncertainty, they still highlight the scale of challenge for agriculture under future climate scenarios.

A number of plant characteristics, including floral growth, pollination, photosynthetic capacity, seedling growth, vegetative features, length of root and shoot, and grain filling, are negatively impacted by drought, which is the leading cause of severe yield losses in agriculture. Drought is considered one of the leading causes of substantial yield loss in global agriculture because it negatively affects a broad range of plant traits, including floral development, pollination, photosynthetic capacity, seedling establishment, vegetative growth, root and shoot elongation, and grain filling [31,32,33,34]. Drought stress induces extensive morphological, physiological, molecular, and biochemical changes (Figure 1) [35]. Although the exact response depends on species, genotype, and stress timing and intensity, the overall pattern of reduced growth and yield under prolonged water limitation is very well-documented across major cereal and legume crops.

At the metabolic level, drought-induced dehydration disrupts numerous processes. Key functions such as carbohydrate metabolism, respiration, chlorophyll content maintenance, photosynthetic electron transport chain, and carbon assimilation, as well as nutrient uptake and translocation, are all sensitive to declines in tissue water potential [36,37]. In major crop species such as barley, rice, wheat, and maize, these disruptions have been extensively studied, with particular emphasis on drought during the reproductive phase, which is consistently identified as the most yield-sensitive stage [18,34,38]. This phase-specific vulnerability is now considered a critical bottleneck for climate-resilient crop production.

A central biochemical consequence of drought is the overproduction of reactive oxygen species (ROS) and related free radicals, including hydrogen peroxide, superoxide radicals, and hydroxyl radicals [39]. Plants with elevated ROS levels experience lipid peroxidation, membrane destabilization, and loss of integrity, as well as oxidative damage to proteins, lipids, and nucleic acids, thereby compromising cellular homeostasis. In parallel, drought and other abiotic stresses stimulate changes in phytohormone profiles; for example, drought stress frequently leads to increased ethylene production. While low to moderate ethylene levels can participate in acclimation of signaling, sustained high ethylene concentrations are typically detrimental, promoting premature senescence, chlorosis, and leaf abscission [40,41].

Presenting strong and well-substantiated negative impacts of drought on plant metabolism, growth and development, and yield, and the additional pressure from increase in global population and rising food demand, enforces that tackling drought stress has become a central priority to maintain food security. Conventional approaches, such as breeding for drought-tolerant cultivars and improving the irrigation efficiency always remain crucial, but they are unlikely to be sufficient on their own in many water-limited regions. In this context, interest is growing to explore biological solutions for complementing agronomic and genetic strategies. Among these, efforts are increasingly being directed towards using AMF to maximize plant and soil functional potential under drought. AMF have the capacity to influence plant water and nutrient acquisition, modulate hormonal and redox status, and improve soil structure, making them promising partners for mitigating the negative impacts of drought stress and enhancing agricultural productivity in an environmentally friendly manner [37].

## 3. AM Fungi Strategies for Alleviating Drought Stress

**Figure 2:**
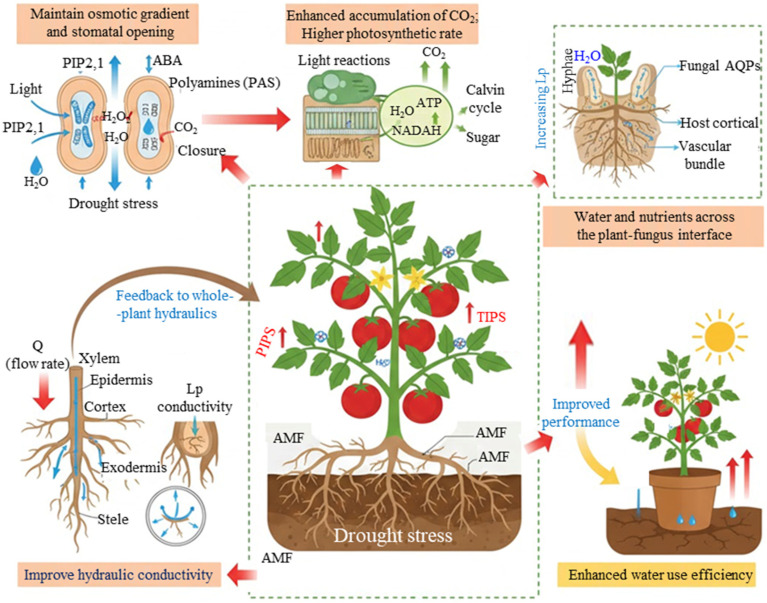
Mycorrhizal symbiosis and drought stress collaborate to regulate aquaporin (AQP) expression, which improves plant stress tolerance. By upregulating aquaporin genes and altering aquaporin proteins, arbuscular mycorrhizal fungi (AMF) help plants better tolerate drought-induced water deficits. The increase in aquaporin activity caused by AMF promotes effective substrate exchange, improves nitrogen uptake at the plant-fungal interface, and increases the hydraulic conductivity of roots. In addition, AMF maintains osmotic gradients, controls stomatal opening, and improves water-use efficiency (WUE) via enhancing transcellular water conduction [6,47]. The direction of the arrows shows the movement of different molecules and particles, more specifically the upward and downward arrows show increasing and decreasing responses.

Drought stress poses a serious threat to global agriculture by degrading soil quality, limiting plant growth, and ultimately reducing crop yields. In this context, AMF have emerged as a major focus of recent research as a symbiotic, biological-based mechanism to shield crops against water limitation. Numerous studies at molecular, physiological, and morphological levels have provided information about the role that AMF can modulate plant drought responses through multiple, interacting pathways (Figure 2) [6,18,42]. Plants themselves employ a suite of strategies to cope with drought, commonly grouped under drought escape, avoidance, and tolerance. A key adaptation is drought escape or avoidance where plants increase water uptake and limit water loss [43,44]. Plants accumulate a range of compatible solutes, including soluble sugars, potassium, calcium, glycine betaine, proline, organic acids, and other low-molecular-weight osmolytes, that lower cytoplasmic osmotic potential and promote water uptake and retention [45,46]. These osmotic adjustment mechanisms help maintain turgor and sustain basic metabolic activity under reduced soil water availability, thereby increasing plant’s resistance to drought and improving its ability to function under water constraints.

Drought activates plant antioxidant defense systems in response to ROS accumulation [48] by upregulating enzymatic and nonenzymatic antioxidants. Because of this, plants can limit oxidative damage to membranes and macromolecules and maintain cellular homeostasis and avoid oxidative burst, a critical component of plants’ response to drought stress. By colonizing host plant root systems as endophytic fungi, AMF improve plant defenses against drought stress. Several discoveries have clarified different ways that AMF help plants cope with drought stress (Table 1). Enhancing water absorption, reducing water loss, regulating the accumulation of compatible solutes and antioxidant defense components are among these strategies [25,42]. The symbiotic relationship between AMF and plants therefore has considerable potential to mitigate the impacts of drought stress on agricultural production, providing a viable path towards the development of effective management approaches.

## 4. AM Fungi-Mediated Improvements in Plant Water Status and Aquaporin Regulation

Plant water status and overall physiology are markedly improved by mycorrhizal associations, especially those formed by AM fungi (Figure 2). This improvement is mainly attributed to a great increase in absorptive surface area provided by the fungal hyphae in the soil, and then the ability of AMF to extract water from the soil regions with lower water potential where plant roots can normally access [50,51]. According to Smith et al. [52], the hyphal length density (HLD) associated with AMF-colonized roots can vary greatly, ranging from 1 to more than 100 m/g of soil, reflecting substantial differences among systems and experimental conditions. During colonization, AMF first produce an appressorium before penetrating the root epidermis, followed by the development of intracellular arbuscules or hyphal coils within the root cortical tissues, which serve as key interfaces for resource exchange [53].

In the rhizosphere, AMF can generate extraradical mycelium that extends more than 100 cm away from the root surface, thereby substantially enlarging the effective root soil contact zone and enhancing water uptake under low soil moisture [49,54]. Because hyphae are much finer than roots, they can access a greater proportion of small, water-filled pores, effectively exploiting soil microsites that are physically inaccessible to roots [55,56]. Thus, the spatial pattern of hyphal growth directly influences the accessibility of water-filled pores and the efficiency of water foraging. The mechanism of water uptake by AMF is therefore particularly important in understanding their role in plant drought responses. Extraradical hyphae absorb water from the soil matrix at distal hyphal tips and transport it through the fungal cytoplasm, crossing the plant–fungus interface and entering root cortical cells by passing through the plant membrane systems [40,45]. This hyphal pathway enhances water uptake and can indirectly help limit ROS production under drought by alleviating severe tissue dehydration [25,57]. Because fungal cell walls are relatively hydrophobic, hyphae can facilitate predominantly unidirectional or bidirectional water movement with minimal backflow to the soil; in effect, they serve as “hydraulic highways” for water transport that chiefly support plant transpiration and improve water acquisition from dry soils [58].

Quantitative estimates underscore the potential importance of these hyphal pathways. Depending on the AMF species, water absorption rates by extraradical hyphae range from 0.13 to 1.97 mg H_2_O h^−1^ [44]. Notably, compared with saturated soils, hyphal water absorption rates increase markedly when soil water deficit develops, indicating a stress-responsive enhancement of hyphal function. By proliferating extraradical hyphae, mycorrhizal associations can decrease the resistance to water flow in the soil–plant continuum under severe drought, thereby improving water uptake and root hydraulic conductivity [59,60]. Through this combination of increased absorptive surface, extended extraradical mycelium, and efficient water uptake, AMF play a major role in supporting plant water status. A detailed understanding of these changes is particularly important for enhancing plant water stress tolerance in arid and drought-prone regions [5,61].

At the cellular level, plants respond to drought stress by regulating key physiological processes, notably by modulating the expression of genes encoding aquaporin (AQP) proteins, which facilitate and control water fluxes across cellular membranes. Aquaporins are integral membrane proteins that act as channels for the passive, highly selective movement of water molecules, and in some cases, small solutes, across biological membranes [62,63]. Based on sequence similarity, plant AQPs have been classified into several major subfamilies (e.g., PIPs, TIPs, NIPs, SIPs, and XIPs), each with characteristic of subcellular localizations and functional properties [64,65].

AMF make a significant contribution to improving plant drought resistance by influencing aquaporin-mediated water transport. In many systems, AMF colonization is associated with upregulation of specific aquaporin genes, leading to enhanced membrane water permeability and improved root and whole-plant hydraulics [66,67]. However, the response of AQP gene expression to drought and AMF is not uniform. Chitarra et al. [68], for instance, reported that in tomato plants colonized by *Funneliformis mosseae*, the nodulin 26-like intrinsic protein gene *LeNIP3;1* was overexpressed, whereas *LeTIP2;3* and LePIP1;1 were downregulated in both mycorrhizal and non-mycorrhizal plants (Figure 2). This illustrates that AMF can induce gene-specific and context-dependent changes in AQP expression.

Aquaporins via fungal origin also appear to contribute directly to drought resistance within the symbiosis. Overexpression of the *RiAQPF2* gene in *Rhizophagus intraradices* under drought conditions has been interpreted as evidence that fungal AQPs facilitate water transport within the fungal network and across the symbiotic interface [52,68]. Likewise, aquaporin genes such as *GintAQPF2* and *RcAQP3* in *Glomus intraradices* and *Rhizophagus clarus* have been linked to fungal water transport capacity and symbiotic water relations [67,69]. Under combined AMF colonization and drought stress, host AQP gene expression exhibits a variety of patterns, with certain genes being upregulated, others downregulated, and some showing no change [65,70]. In *Rhizophagus irregularis*, drought-stressed mycorrhizal roots and extraradical mycelia exhibit elevated expression of two AQP genes, *GintAQPF1* and *GintAQPF2*, further supporting a direct role of fungal AQPs in enhancing water transport and tolerance to water deficit [64].

Studies in *Lotus japonicus* and *Zea mays* underscore the complexity of plant–AMF–AQP interactions during drought. In maize, for example, enhanced drought tolerance in AMF-colonized plants has been associated with a broader and differential regulation of AQP-related genes in the roots, including cases where downregulation of particular PIP isoforms may contribute to reduced water loss and improved water-use efficiency under stress (Figure 2) [71]. Water transport is facilitated by the *L. japonicus* gene *LjNIP1*, which is expressed in cortical cells that have arbuscules inside the roots [67,72].

Overall, fungal AQP expression patterns appear to act, at least in part, as a compensatory or complementary mechanism to host AQPs during drought stress, supporting a cooperative role in improving root water uptake and distribution [62,73]. As a result, AMF-colonized plants often experience less severe soil and tissue drying and show reduced oxidative stress compared with non-mycorrhizal plants. However, the diversity of AQP responses reported in the literature also indicates that plant and fungal aquaporins do not respond in a single, uniform way to drought and AMF; instead, their regulation is highly context- and gene-specific.

## 5. AM Fungi-Driver Modulation of Plant Hormones Network Under Drought

The symbiotic interaction between AMF and plants is closely related to sustainable plant growth and development [6,19,74]. A central feature of this relationship is its effects on plant hormone homeostasis, which in turn contributes to the enhanced drought tolerance commonly observed in mycorrhizal plants [47,75]. As AM symbiosis is developed and maintained, the levels and distribution of several key phytohormones, including auxins, gibberellins (GA), salicylic acids (SA), jasmonates, abscisic acid (ABA), and cytokinins, are altered in a tissue- and context-dependent manner [6,76]. Through these hormonal shifts, AMF modulate plant physiology and metabolism, influencing processes such as root architecture, shoot growth, nutrient allocation, and defense responses [77,78]. In many cases, mycorrhizal plants exhibit higher concentrations of growth-promoting hormones than their non-mycorrhizal counterparts [79,80], although the magnitude and direction of hormonal changes can vary depending on the specific fungal isolate and host genotype [81].

The establishment of AM symbiosis involves the rerouting of nutrients to the fungus-colonized root tissue, the development of fungal structures (e.g., arbuscules and vesicles), and the regulation of plant growth patterns under the impact by mycorrhizal signals. Plant hormones are deeply integrated into these processes. They regulate the balance between root and shoot growth, influence the formation and turnover of arbuscules, and coordinate plant defense and stress responses in the presence of AMF [6,54,82]. Thus, hormone signaling acts as a key interface linking the nutritional and developmental roles of AMF with the plant’s capacity to withstand drought stress.

Among these hormones, ABA is one of the plant hormones that has been extensively studied in relation to AM symbiosis and drought. ABA is widely recognized as a central component of plant stress signal transduction and a critical regulator of drought responses [83,84,85]. In the context of AMF, ABA has been implicated both in regulation of arbuscules development and function, and in determining the degree of plants’ responsiveness or susceptibility to colonization [6,45,80]. By regulating stomatal closure and root hydraulic properties, ABA plays a dual role, such as contributing to the establishment and performance of the AM symbiosis, and at the same time, governing how plants control water loss and uptake under soil water deficit (Figure 2). In particular, ABA influences transpiration rate and root hydraulic conductivity, thereby linking soil moisture status to whole plant-water relations [44,86,87].

However, the direction of ABA changes in mycorrhizal plants is not uniform. Studies under non-stress conditions have sometimes reported higher ABA levels in mycorrhizal plants, whereas under drought, ABA levels in AMF plants can be lower, similar, or higher than in non-AM controls, depending on species, tissue sampled and timing of measurement [45,88]. These apparently contradictory results suggest that AMF do not simply increase or decrease ABA in a linear way; instead, they seem to contribute to a more refined regulation of ABA dynamics, allowing plants to optimize the balance between root water uptake and leaf transpiration. This interpretation is supported by experiments with exogenous ABA, where AM and non-AM plants often respond differently to ABA application under drought. In these cases, AM plants tend to maintain a more favorable combination of root water transport capacity and stomatal regulation, which translates into improved water status and drought tolerance [44,89]. The importance of ABA in AM symbiosis is further supported by the altered expression of ABA biosynthesis and signaling gene in colonized roots [80,90].

The interaction between ABA, plant root hydraulics, and AM symbiosis has been explored in more detail in numerous studies (Figure 2). For example, exogenous ABA application has been shown to increase root hydraulic conductivity in both mycorrhizal and non-mycorrhizal plants, but AM plants may display lower baseline hydraulic conductivity, particularly when ABA levels are high [85]. This somewhat counterintuitive pattern has been associated with the downregulation of certain plasma membrane intrinsic protein (PIP) aquaporins, suggesting that AM plants may rely on tighter control of membrane water transport to maintain internal water status. In practical terms, this may allow AM plants to sustain higher relative water content in shoots by limiting excessive water loss while still benefiting from improved soil water access via the fungal network. Overall, ABA appears to be necessary not only for the long-term establishment and stability of AM colonization [85,91], but also for fine-tuning plant hydraulic responses during drought within the mycorrhizal context.

## 6. AM Fungi Regulation of Plant Antioxidant Systems

When plants are exposed to drought stress, their metabolism is profoundly reprogrammed, and multiple pathways can become uncoupled. One major consequence is the diversion of high-energy electrons from photosynthetic electron transport to molecular oxygen, resulting in the overproduction of ROS. Under optimal conditions, when CO_2_ concentrations are saturating, and stomata remain open, the fraction of electron flow “escaping” to oxygen is negligible [37,92]. Nonetheless, under CO_2_ shortage conditions, such as during drought-induced stomatal closure, up to 50% of the entire photosynthetic electron flux can be diverted to O_2_, greatly enhancing ROS formation [93]. According to Miller et al. [94], ROS, which include single oxygen molecules (^1^O_2_), hydrogen peroxide (H_2_O_2_), superoxide (O_2_^−^), and hydroxyl radicals (HO^.^), are known to be harmful because they can cause oxidative damage to vital biological components like proteins, DNA, and lipids. Paradoxically, these same molecules act as important secondary messengers, triggering signal transduction cascades and acclimatory responses. One of the earliest and ubiquitous responses of plants to drought stress is the rapid generation of ROS [31,34,95]. To prevent oxidative damage while preserving ROS signaling, plants rely on a sophisticated antioxidant defense system composed of both enzymatic and nonenzymatic components.

**Figure 3:**
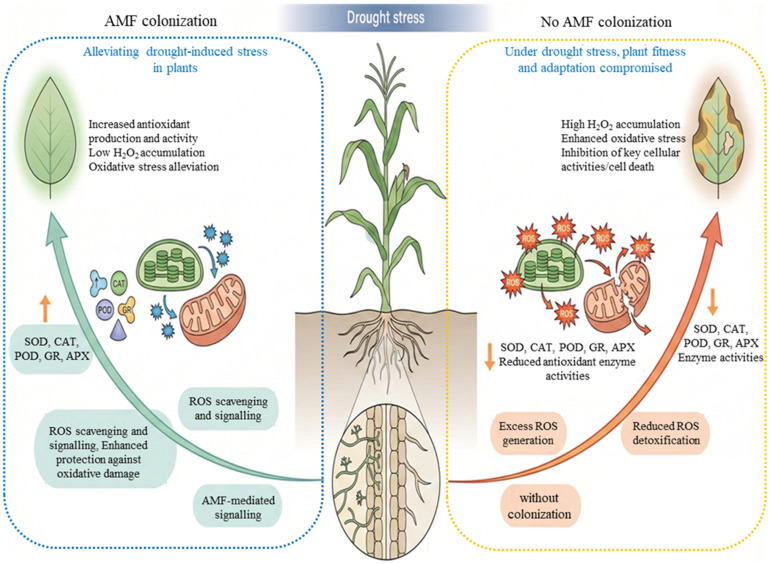
In order to help plants regulate and control a variety of processes and lessen the negative effects of drought stress, the arbuscular mycorrhizal fungi (AMF) symbiosis is essential. This support comes from interactions that affect plant growth performance, either directly or indirectly [6]. The upward and downward arrows show the increasing and decreasing responses.

It is commonly acknowledged that enzymatic antioxidants are considered the primary barrier against ROS overaccumulation (Figure 3). Superoxide dismutase (SOD) catalyzes the dismutation of O_2_^−^ to H_2_O_2_, which is then further detoxified by enzymes such as glutathione reductase (GR), catalase (CAT), guaiacol peroxidase (G-POD), dehydroascorbate reductase, monodehydroascorbate reductase, and ascorbate peroxidase (APX) [96,97]. These enzymes are tightly embedded in redox cycles (e.g., the AsA–GSH cycle), allowing continuous regeneration of antioxidant pools. Complementing these enzymatic defenses, nonenzymatic antioxidants also play a major role in ROS scavenging. In order to play crucial roles in neutralizing ROS, carotenoids quench singlet oxygen and lipid radicals, while low molecular weight thiols such as glutathione (GSH), tocopherol and ascorbic acid (AsA) directly neutralize ROS and support redox buffering [80,98]. The dynamic balance between ROS production and the combined capacity of enzymatic and non-enzymatic antioxidants is therefore a key determinant of plant performance under drought. A mechanistic understanding of how these systems are regulated, and how they interact with other drought-response pathways, underpins the development of further strategies to enhance drought resistance.

Within this oxidative framework, the interaction between AMF and their host plants emerges as a critical factor in modulating antioxidant defenses, especially under drought stress. Numerous studies have reported that AMF-colonized plants exhibit a reduced oxidative burst, higher antioxidant enzyme activities, and lower accumulation of oxidative damage markers compared with nonmycorrhizal plants [6,96]. A quantitative synthesis by Lokhandwala and Hoeksema [99] suggests that, on average, plants linked to AMF have an estimated 16% increase in the production of antioxidant enzymes, although the magnitude of this effect is highly variable and depends on the particular host–fungus combination and environmental context. Thus, while the overall trend towards enhanced antioxidant capacity in AMF plants is well-supported, the specific responses and effect sizes are context-dependent rather than universal.

Numerous studies in crops provide detailed examples of these general patterns. In maize, AMF symbiosis has been shown to reduce nonsystematic oxidative damage in drought-stricken plants by lowering ROS levels and enhancing antioxidant activity [100]. In sesame, dual inoculation with *F. mosseae* and *R. irregularis* markedly increased the activities of APX, polyphenol oxidase, G-POD, CAT, and phenylalanine ammonia-lyase (PAL) under severe drought conditions, and this was accompanied by a concurrent rise in total soluble sugar concentration (Figure 3) [101]. Mycorrhizal tea plants similarly displayed elevated activities of SOD, CAT, APX, and G-POD, as well as increased expression of antioxidant-related genes (CsSOD and CsCAT), demonstrating a greater capacity to mitigate oxidative damage under water deficit [97]. In saffron, AMF colonization influenced both antioxidant and nitrogen-assimilating enzymes, including that AMF-mediated redox adjustment is intertwined with nitrogen metabolism [102]. Likewise, chicory plants associated with *R. irregularis* exhibited enhanced antioxidant defense mechanisms, including SOD, POD, AsA, and GSH, which were linked to reduced H_2_O_2_ accumulation and lower oxidative damage [103].

These physiological and biochemical changes are supported by transcriptional and metabolic reprogramming. Increased transcription levels of genes encoding antioxidant enzymes and enzymes involved in AsA and GSH biosynthesis have been reported in AMF-inoculated plants under drought stress [104,105]. Such gene-level evidence suggests that AMF regulation of antioxidant capacity operates not only through post translational activation of existing enzymes but also through the more upstream control of gene expression. In lettuce, AMF colonization led to higher accumulation of antioxidant metabolites, particularly carotenoids and anthocyanins, with these effects being more pronounced under water deficit than under well-watered conditions [56,106]. Together, these findings indicate that AMF can simultaneously modulate enzymatic and non-enzymatic antioxidant pools, thereby contributing to a more robust redox buffering capacity in drought-stressed plants [57,107].

High-throughput transcriptomic analyses further reveal that AMF-mediated oxidative stress mitigation involves broader metabolic adjustments. RNA sequencing has shown that AMF can activate multiple pathways associated with redox homeostasis, including the pentose phosphate pathway, which generates NADPH for antioxidant reactions, as well as pathways linked to methionine and sulfur metabolism and the upregulation of genes encoding antioxidant enzymes [108]. These integrated changes highlighted that AMF symbiosis influences redox balance at several regulatory levels—from metabolism to gene expression—and helps explain why mycorrhizal plants often show enhanced drought tolerance compared with non-mycorrhizal controls.

The available evidence supports the conclusion that AM symbiosis plays a critical role in reducing oxidative stress and increasing plant resistance to drought. As emphasized by Bartels [109], plants can effectively deal with drought conditions by avoiding excessive oxidative stress and efficiently detoxifying ROS, AMF contribute to both aspects by enhancing antioxidant capacity and modulating the magnitude and timing of ROS production. Recent studies confirm that plants inoculated with AMF typically display higher expression levels of genes encoding antioxidant enzymes (Figure 3), which increases their ability to maintain ROS homeostasis under drought [80,110]. At the same time, AMF-mediated improvements in plant water status and drought avoidance can reduce ROS formation at the source, meaning that in some cases, lower ROS production may obviate the need for very strong upregulation of antioxidant enzymes. This idea is supported by studies in which AMF plants show reduced oxidative markers without proportionally large increases in antioxidant activity [14,111,112].

The magnitude, direction, and even presence of antioxidant responses to AMF, however, are not uniform across all plant-fungus combinations. For instance, *R. intraradices* and *F. mosseae* have been reported to improve essential oil content and increase drought tolerance in *Salvia yangii* and *Salvia abrotanoides*, with these benefits partly attributed to changes in antioxidant activity and secondary metabolism [52]. Marulanda et al. [104] observed that autochthonous *Glomus* species outperformed allochthonous strains in increasing growth, water content, and nutrient uptake in drought-stressed *Lavandula spica*, suggesting that local adaptation of AMF communities can influence the strength of antioxidant-related benefits. Research on *Diversispora spurca* also showed activation of antioxidant defense systems, such as ASC-GSH cycle and other enzymatic mechanisms, supporting the growth of walnut plants under drought [113]. A meta-analysis by Chandrasekaran and Paramasivan [54] further concluded that AMF are generally effective in reducing H_2_O_2_ through upregulation of antioxidant enzymes, but also emphasized that the magnitude and consistency of this effect depend strongly on both AMF identity and host plant species under drought stress. Thus, while the direction of AMF effects on antioxidant defenses is often positive, the variability among systems and methodological differences across studies mean that responses cannot be assumed to be universally large or uniform.

## 7. AM Fungi Effects on Soil Properties, Structure, and Water Retention

In the context of mitigating drought, the effects of AMF on soil water retention and hyphal density are critical. As highlighted by Geng et al. [114], drought stress in agricultural soils arises not only from reduced rainfall but also from inadequate water retention capacity, poor nutrient and water percolation, and excessive surface runoff. Because AMF can boost soil fertility, nutrient absorption, and development, and soil retention under drought, they have gained prominence as key biological allies in alleviating plant stress [6]. Mycorrhizal effects on plant water relations under drought are often linked to changes in soil water retention characteristics. For example, mycorrhization by *G. intraradices* in *Sequatchie loam* increased extraradical hyphal density and the proportion of water-stable aggregates, which in turn improved soil moisture retention. These findings support the notion that mycorrhizal symbiosis can improve plant drought resistance partly by modifying soil structure and hydraulic behavior.

Moisture levels also modulate plant WUE in the presence of AMF (Figure 2). By extending into a wider rhizosphere volume and exploiting smaller soil pores, fungal hyphae enhance plant water status under both well-watered and water-limited conditions [16,46]. When inoculated into crops, several AMF species, including *Glomus mosseae*, *G. claroideum*, *G. intraradices*, and *G. coronatum*, have been shown to significantly increase water uptake compared with non-mycorrhizal controls [46,115]. These effects are closely tied to improvements in soil structure; interactions between extraradical mycelium and soil particles promote the formation of stable aggregates, which help retain and reduce erosion [116,117]. AMF further promotes carbon sequestration and the development of solid aggregates by releasing hydrophobic organic materials, such as polysaccharides, glomalin-related soil protein (GRSP), and mucilage, into the soil matrix [6,118]. Quantitatively, various pot and field studies have reported that AMF inoculation can increase GRSP concentrations by roughly 20–50% and the proportion of water-stable macroaggregates by about 15–40% relative to non-mycorrhizal controls, changes that are substantial enough to measurably improve soil water infiltration and retention under drought conditions [113]. Although much of the evidence for GRSP is correlative, these consistent associations strongly suggest that GRSP is an important mediator of aggregate stability and contributes to plant adjustment to osmotic stress in the soil.

Stomatal traits provide another interface between AMF-mediated soil effects and plant water loss. Stomatal size and density are key determinants of stomatal conductance and transpiration. Reductions in stomatal size are often considered a structural component of drought adaptation, whereas stomatal density can respond more flexibly to environmental cues. The observed impacts of AMF on stomatal conductance in both host and non-host plants may reflect not only direct plant fungus signaling but also indirect effects mediated through changes in rhizosphere processes soil biota [97,99,119]. By improving water and nutrient uptake, AMF can improve stomatal regulation and mitigate the negative impacts of drought on gas exchange and photosynthesis [43,99]. For instance, increased stomatal conductance and better leaf water status have been reported in AMF-inoculated *Poncirus trifoliata* and *Rosmarinus officinalis* under drought stress, illustrating the beneficial effects of AM symbiosis on leaf physiology [77,120]. In turn, more effective water exploration by mycorrhizal fungi can intensify wet–dry cycles in the soil, which can further promote aggregate formation and stabilization [121].

AMF-plant interactions are crucial to improve soil water retention, aggregate stability, nutrient availability, and stomatal control, all of which contribute to enhanced drought tolerance. The mechanisms suggested that AMF-mediated drought tolerance in crops emerges from a tightly interconnected network spanning the root, shoot, and soil environment, rather than from any single process in isolation. At the root cellular level, AMF alter the expression and activity of host and fungal aquaporins, thereby modulating radial and axial water transport pathways and plant hydraulic conductivity [56]. These hydraulic adjustments are coordinated with changes in hormonal signaling, particularly ABA and its interaction with other growth- and defense related hormones, which integrate soil water status with stomatal behavior and root growth responses [6]. In parallel, AMF prime and enhance antioxidant systems that help maintain ROS homeostasis under water deficit, as shown by both individual experiments and meta-analytical syntheses of antioxidant enzyme responses [50,89]. These within-plant processes are further supported by AMF-driven improvements in soil structure, aggregate stability, and water retention, which influence the availability and spatial distribution of water and nutrients around roots. Figure 3 presents a conceptual model that integrates these aquaporin, hormone, antioxidant, and soil mediated processes into a unified framework, illustrating how AMF can collectively enhance crop drought tolerance at multiple scales relevant to sustainable agriculture.

## 8. Translating AM Fungi into Drought-Resilient Agricultural Production

AMF represent a promising, biologically based tool to alleviate drought stress and sustain crop yields in water limited production systems (Table 1). By forming mutualistic symbiotic associations with the roots of most crop species, these fungi establish a bidirectional resource-exchange interface through which carbon from the host is traded for mineral nutrients, water, and stress-modulatory services [6,19]. In agricultural settings, AMF mitigate drought through a suite of interconnected mechanisms that collectively enhance plant resistance to water deficit rather than via a single pathway in isolation.

For agricultural systems, AMF improves the absorption of vital nutrients, especially phosphorus [1,4]. Crop plants’ ability to take up nutrients from the soil is often impaired when they are under drought stress. AMF can efficiently explore a larger soil volume, thanks to their extensive hyphal network, which improves nutrient extraction and efficient transport to the host crop plant [16,17]. Agricultural fields may be able to sustain physiological functions even in the face of water scarcity because to this enhanced nutrient uptake [23]. Beyond the plant root zone, the AMF hyphal network searches the soil for water sources. The crop may absorb more water as a result, particularly in parts of the soil that are harder for the roots of the plant to reach [21]. Furthermore, it has been discovered that the extraradical mycelium of AMF improves soil structure and water retention. This is essential when there is a drought and it is difficult to keep sufficient soil moisture [20]. AMF has the ability to cause plants to express genes that respond to stress, which is crucial for the activation of drought defense mechanisms. This involves the synthesis of antioxidants, osmo-protectants, and other substances that aid the plant in overcoming oxidative stress under drought conditions [22]. Therefore, the symbiotic association with AMF can strengthen the plant’s resistance to oxidative damage under drought conditions. AMF encourages the growth of a more comprehensive and effective crop root system [49]. The plant can obtain water and nutrients from a greater region because of increased root biomass [2]. For plants under drought stress, a strong root system is essential because it enables them to access deeper soil layers where water may be more readily available [5]. AMF can provide a healthy microbial environment by influencing the entire microbial community in the crop rhizosphere [3]. Pathogenic microorganisms may be suppressed, and other helpful soil microbes may be encouraged. Plant resistance to abiotic stresses like drought is indirectly supported by a better soil ecology, which is a result of a balanced microbial community [14].

It is crucial to take into account elements like choosing appropriate AMF strains, refining inoculation techniques, and comprehending the interactions between AMF and other soil bacteria in order to fully utilize AMF’s potential for drought mitigation in crop production [15,17]. To confirm the efficacy of AMF-based treatments under various environmental circumstances and agricultural practices, field trials and on-farm investigations are crucial. Furthermore, research ought to concentrate on creating economically feasible AMF inoculants for broad use in agriculture, giving farmers an affordable and long-lasting way to lessen the negative effects of drought on crop production.

## 9. Challenges and Future Directions in the Study of AM Fungi for Crop Drought Mitigation

One promising approach to environmentally friendly and sustainable farming methods is the use of AMF to help crops withstand drought. Although the beneficial roles of AMF in promoting plant growth and mitigating drought stress have been recognized, several critical challenges still limit their reliable and large-scale application in agriculture. Addressing these challenges requires not only recognizing the potential of AMF, but also clearly identifying the main gaps in current knowledge and defining research priorities that can guide future work.

A first major challenge is ensuring the effective and stable colonization of crop roots under field conditions. While many studies demonstrate high levels of AMF colonization and clear benefits in controlled environments, much less is known about how inoculated AMF strains compete with diverse native soil microbiota and persist over time in agricultural fields. A key knowledge gap is how different AMF species and strains perform across a range of soil types, climates, and management practices. Future research should therefore prioritize selecting and characterizing AMF strains that exhibit high colonization competence and persistence in realistic agroecosystems, and testing how these strains interact, synergistically or competitively, with indigenous fungal communities and other root associated microbes.

A second, closely related challenge lies in the strong influence of soil properties and environmental context on AMF performance. Soil texture, structure, pH, nutrient availability (particularly nitrogen and phosphorus), and organic matter content can all modulate the effectiveness of AMF-based interventions. At present, there is limited mechanistic understanding of how specific soil characteristics constrain or enhance AMF establishment, hyphal development, and functional contributions under drought. Research priorities here include systematic evaluations of AMF strains across contrasting soil types and fertility levels, and experiments that explicitly manipulate key soil variables (such as pH or N:P ratios) to determine thresholds and optimal ranges for AMF-mediated drought mitigation.

A third major gap is the translation of promising findings from laboratory and greenhouse studies into robust field applications. Many demonstrations of AMF-induced drought tolerance are based on short-term pot experiments with controlled stress levels, which may not reflect the temporal variability, heterogeneity, and multiple stress factors encountered in the field. To guarantee successful and predictable incorporation of AMF into agricultural practices, research must focus on refining application techniques, such as inoculum formulation, dose, timing, and mode of delivery (e.g., seed coating, in-furrow application, or transplant dipping), and testing them under long-term, multi-site field trials. Such trials should be designed to quantify not only plant growth and yield, but also cost–benefit ratios and interactions with existing management practices, including fertilization, irrigation, tillage, and crop rotation.

A fourth priority concerns the mechanistic understanding of how AMF confer drought resistance at molecular, physiological and whole-plant scales. Although numerous individual studies have described changes in signaling cascades, gene expression and biochemical pathways in AMF-colonized plants, these processes are often examined in isolation. There is still a need to integrate knowledge about aquaporin regulation, hormonal networks, antioxidant systems and carbon allocation into cohesive models that explain when and why AMF provide strong benefits under drought. Future research should therefore focus on dissecting these signaling and regulatory networks using combined approaches (e.g., transcriptomics, metabolomics, and targeted physiological measurements), and on identifying key regulatory nodes or traits that could be used to screen and select both crop genotypes and AMF strains for improved drought resilience.

While the potential benefits of AMF are significant, challenges remain:

Variability: Different AMF species have varying effects on plant species, necessitating further research to understand specific interactions.

Field Application: Translating laboratory findings to field conditions can be complex due to soil type, microbial communities, and environmental factors.

Management Practices: Developing effective management practices that promote AMF activity requires a better understanding of agricultural inputs, practices (e.g., tillage), and soil health.

The integration of molecular insights into the role of arbuscular mycorrhizal fungi can enhance their applications in agriculture, promoting sustainable practices that improve plant growth, resilience, and soil health. Continued research into AMF functions and interactions will be critical for optimizing their use in agronomic systems, particularly in the context of climate change and the need for sustainable agricultural practices.

Finally, AMF effectiveness is strongly shaped by interactions with other rhizosphere organisms and environmental factors, yet these interactions remain underexplored. Temperature, water availability, co-occurring beneficial microbes (such as rhizobia and plant growth-promoting rhizobacteria), and potential pathogens can all influence the outcome of AMF–plant symbioses under drought. Future studies should explicitly address how AMF function within complex rhizosphere communities and under fluctuating environmental conditions, including combined stresses (e.g., drought with heat or salinity). This will help identify compatible microbial consortia and management strategies that maximize the positive contributions of AMF while minimizing potential trade-offs.

## 10. Conclusions

The world’s climate is changing more rapidly than ever before, exposing plants and their symbiotic partners to increasingly frequent and intense abiotic stresses. These changes have profound direct and indirect effects on plant growth and productivity, and the stability of agroecosystems. AM fungi can enhance host-plant tolerance to drought stress, but the reciprocal effects of drought on AM fungi themselves, and on the long-term functioning of the symbiosis, are still not fully understood.

Drought stress can act directly on AM fungi, altering their fitness, diversity, community composition, and symbiotic efficiency, or indirectly via changes in host physiology, carbon supply, and root architecture. Under current and projected climate scenarios, especially in relation to drought, it becomes essential not only to study how AM fungi improve plant performance, but also to understand how drought affects AM fungi independently of the host. Such knowledge will clarify both the advantages and limitations of this ubiquitous symbiosis and will be critical for designing robust strategies to apply AM fungi-based technologies in sustainable agriculture.

The economical production of viable, effective AM fungi inocula for use as biofertilizers offers considerable promise in this context, yet remains constrained by limited research capacity, technical challenges, and inconsistencies in field performance. Overcoming these bottlenecks will require advances in strain selection, inoculum formulation, quality control, and compatibility with different cropping systems.

**Figure 4:**
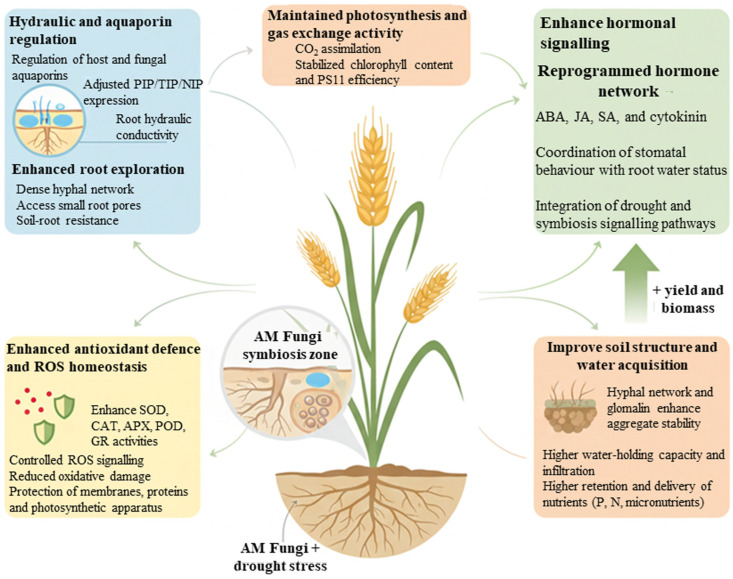
Integrated conceptual model summarizing how AM fungi enhance crop performance under drought stress. AM fungi colonization enhances root hydraulics and exploration, reprograms hormone and antioxidant networks, and improves soil structure and water and nutrient acquisition. Together, these coordinated processes help maintain photosynthesis and gas exchange under drought, ultimately sustaining crop biomass and yield.

To achieve sustainable agricultural production and ensure food security, AM fungi need to be explored and exploited at multiple levels, from genes and physiological traits to field-scale management practices, in diverse environments, where crops face drought stress simultaneously. A deeper understanding of how AM fungi interact with aquaporins and plant hydraulics, hormonal signaling networks, antioxidant systems, and soil physical properties, as synthesized in this review, can guide the development of more targeted strategies to enhance crop drought resistance (Figure 4). At the same time, ensuring the stability and efficacy of AM fungi-based bioinoculants in real field conditions remains a central priority. In this regard, innovative technologies such as nanoencapsulation and improved carrier systems offer promising avenues to increase the shelf life, delivery efficiency, and colonization success of AM fungal inocula, particularly in drought-prone regions. Such advances could lead to new formulations that maximize the establishment, spread, and functional expression of beneficial mycorrhiza on host plants.

During drought, AM fungi influence not only plant performance but also key soil processes. Their effects on soil structure, nitrogen and phosphorus cycling, soil microbial communities, and overall soil health must be examined more thoroughly, including through molecular and ecological approaches. A better grasp of “rhizosphere engineering” under drought, how AM fungi, roots, and associated microbiota collectively shape the soil environment, will be central to designing comprehensive strategies for resilient, low-input agriculture.

## Figures and Tables

**Table 1:** Overview of how AM fungi enhance crop performance under drought stress in different species, summarizing key responses reported from pot and field experiments.

Pot Experiments				
Plant Species	AMF Species	Method/Stress Type	Plant Performance	Reference
*Zea mays*	*Rhizophagus irregularis* SUN16 *Glomus monosporum* WUM11	Well-watered (80% soil moisture content), Moderate drought (60%), Severe drought (40%)	Root colonization^↑^, Glomalin contents, Microbial biomass^↑^, Nutrient uptake^↑^, Antioxidant activity^↑^, Photosynthetic efficiency^↑^, Water use efficiency^↑^	[49]
*Triticum monococcum* *Triticum dicoccum* *Triticum aestivum*	*Funneliformis mosseae*	80% (well-watered) and 40% (drought stress) field water capacity	Leaf area^↑^, Photosynthetic rate^↑^, Stomatal conductance^↑^, Water use efficiency^↑^	[2]
*Triticum aestivum* L.	AMF spores (*Glomus* sp. and *Gigaspora* sp.)	Control: plant irrigated well (90% field capacity) Drought (70% field capacity)	Chlorophyll concentrations^↑^, PSII^↑^, RWC%^↑^, Growth^↑^, Grain yield^↑^	[3]
*Annona muricata* L.	*Rhizophagus intraradices Funneliformis mosseae*	For the irrigation factor, there were two levels: normal (field capacity, RN) and low (half field capacity, RB). Plants under RN were watered to field capacity every 7 days (61% substrate moisture), while plants under RB were watered every 14 days (29% substrate moisture), with 1 L of water applied.	Plant height^↑^, Stem diameter^↑^, Leaf number^↑^	[5]
*Zea mays* L. cultivar B73	*Funneliformis mosseae*	Well-watered and water stress treatments were watered to 80% and 35% soil moisture content	Maize seedling growth^↑^, Plant biomass^↑^, Chlorophyll content^↑^, Antioxidant capacity^↑^, Soil nutrient availability^↑^, Microbial biomass^↑^	[14]
*Populus cathayana*	*Rhizophagus irregularis*	Control and drought stress	Chlorophyll fluorescence parameters^↑^, Heme synthesis^↑^, 5-aminolevulinic acid (ALA) pathway^↑^, Proline^↑^, Pipecolic acid^↑^, and α-aminoadipate^↑^	[15]
*Zea mays* L. (Zhengdan 958)	*Acaulospora scrobiculata* *Paraglomus occultum* *Rhizophagus intraradices* *Glomus versiforme* *Funneliformis mosseae* *Claroideoglomus etunicatum*	Water treatments consisted of drought stress (maintained at 40 ± 5% of field capacity) and well-watered conditions (maintained at 80 ± 5% of field capacity).	Shoot biomass^↑^, Root biomass^↑^, Plant height^↑^, Leaf area^↑^, Root length^↑^, Root surface area^↑^. Superoxide dismutase^↑^, Catalase activities^↑^, Glutathione^↑^, Ascorbic acid contents^↑^ in roots; and Peroxidase^↑^, Catalase activities^↑^ and Glutathione content^↑^ in leaves. Indoleacetic acid^↑^, Ethylene contents^↑^ in roots and leaves.	[4]
*Leymus chinensis*	*Funneliformis mosseae* *Claroideoglomus etunicatum*	Three drought treatments (no drought (75.00% field capacity), mild drought (56.25% field capacity), severe drought (37.50% field capacity)	Key biochemistry parameters^↑^, Soluble sugar concentration^↑^, Antioxidant enzyme activities^↑^, Plant productivity^↑^, Photosynthetic activity^↑^	[17]
*Vicia faba* L.	*Rhizophagus irregularis*, *Gigaspora margarita, Funneliformis mosseae*, *F. constrictum*	1. Well-watered: Plants were irrigated with 90% water holding capacity. 2. Drought stress (DS: 30%): Plants were irrigated with 30%.	Growth parameters^↑^, Cellular hydration^↑^, Activity of antioxidant enzymes^↑^ (Superoxide dismutase^↑^, Catalase^↑^, Peroxidase^↑^, Ascorbate peroxidase^↑^, Polyphenol oxidase^↑^), Organic adjustments^↑^, Total soluble protein^↑^, Proline^↑^, Total soluble carbohydrate^↑^, Soil-rich glomalin content^↑^, both easily and total extractable.	[16]
*Triticum aestivum* L.	*Rhizophagus irregularis*	Well-watered (80% field water capacity), moderate water stress (50%), and severe water stress (35%)	Soil organic carbon^↑^, Microbial biomass carbon^↑^, Particulate organic carbon^↑^, Oxidizable carbon^↑^, Dissolved organic carbon^↑^, Carbon sequestering enzymes^↑^ (Xylosidase^↑^, β-Glucosidase^↑^, and cellobiohydrolase^↑^), Carbon emission efficiency^↑^, CO_2_ assimilation^↑^, Net carbon balance^↑^, Water use efficiency^↑^, Grain yield^↑^	[1]
**Field experiments**				
*Glycine max*	*Funneliformis mosseae Rhizophagus intraradices Claroideoglomus etunicatum*	Irrigation after 20% (optimal irrigation), 50% (moderate water stress), and 80% (severe water stress) soil moisture depletion.	Nitrogen^↑^, Phosphorus^↑^, Potassium, and Zinc uptake^↑^, Proline^↑^, Soluble carbohydrates^↑^, Ascorbate peroxidase^↑^, Guaiacol peroxidase^↑^, Catalase^↑^, Linoleic and linolenic acid concentrations^↑^, Seed yield^↑^, Oil content^↑^, Iodine value^↑^	[23]
*Zea mays*	*Rhizophagus irregularis SUN16 Glomus monosporum WUM11*	The mulching treatments consisted of plastic film mulching (PFM) and no mulching (non-PFM). No irrigation.	Plant signaling hormones^↑^, Photosynthetic pigments^↑^, Photosynthesis^↑^, Nutrient translocation^↑^, Nitrogen^↑^, Phosphorus^↑^, Potassium^↑^, Biomass accumulation^↑^	[22]
*Camelina sativa*	*Funneliformis mosseae Claroideoglomus etunicatum Rhizophagus irregularis*	Three irrigation regimes: (i) full irrigation throughout the growing season (IR1), (ii) limited irrigation from flowering (IR2), and (iii) limited irrigation from pod formation (IR3).	Seed and oil yield^↑^, Chlorophyll fluorescence^↑^, Nutrient availability^↑^, Phenolics^↑^, Flavonoids^↑^, Ascorbic acid^↑^, Proline^↑^, Soluble sugars^↑^, Tocopherols^↑^. Mycorrhizal colonization^↑^, Enzymatic antioxidant defenses^↑^, Unsaturated fatty acids^↑^	[20]
*Cichorium pumilum* Jacq.	*Glomus mosseae* *Glomus intraradices* *Glomus etunicatum*	Experimental factors were consisted of three irrigation regimes based on percentage of total available water capacity (AWC): (i) 55% AWC (well-watered control), (ii) 35% AWC (moderate drought), and (iii) 20% AWC (severe drought) as the main plot	Proline^↑^, Soluble sugar^↑^, Chlorophyll a, b, and total Chlorophyll^↑^, Carotenoids^↑^, Plant growth^↑^, Yield components^↑^, Fruit yield^↑^, Inulin^↑^	[21]

^↑^ indicate increasing responses.

## Data Availability

No new data were created or analyzed in this study. Data sharing is not applicable to this article.

## Abbreviations

The following abbreviations are used in this manuscript:

ABA	Abscisic Acid
AMF	Arbuscular Mycorrhizal Fungi
AQP	Aquaporin
ATP	Adenosine Tri-Phosphate
WUE	Water-Use Efficiency
SOD	Superoxide dismutase
CAT	Catalase
G-POD	Guaiacol Peroxidase
POD	Peroxidase
GR	Glutathione Reductase
APX	Ascorbate Peroxidase
ROS	Reactive Oxygen Species
H_2_O_2_	Hydrogen Peroxide
SA	Salicylic Acids
AsA	Ascorbic Acid
GSH	Glutathione
GA	Gibberellins
CO_2_	Carbon dioxide
CH_4_	Methane
HLD	Hyphal Length Density
PIPs	Plasma Membrane Intrinsic Proteins
TIPs	Tonoplast Intrinsic Proteins
NIPs	NOD26-like intrinsic proteins
SIPs	Small Basic Intrinsic Proteins
XIPs	Plant-Specific Subfamily of X-Intrinsic Protein
*LeNIP3;1*	Lycopersicon esculentum Nodulin 26-like Intrinsic Protein 3;1
*LeTIP2;3*	*Lycopersicon esculentum* Tonoplast Intrinsic Protein 2;3
*LePIP1;1*	*Lycopersicon esculentum* Plasma membrane Intrinsic Protein 1;1
*RiAQPF2*	*Rhizophagus irregularis*/*Rhizophagus intraradices* Aquaporin Family 2
*GintAQPF2*	*Glomus intraradices* Aquaglyceroporin Family 2
*RcAQP3*	*Rhizophagus clarus* Aquaglyceroporin 3
*LjNIP1*	*Lotus japonicus* Nodulin-26-like Intrinsic Proteins 1
DNA	Deoxyribonucleic acid
PAL	Phenylalanine Ammonia-Lyase
*CsSOD*	*Cymbidium sinense* Superoxide Dismutase
*CsCAT*	*Cucumis sativus* Catalase
RNA	Ribonucleic Acid
NADPH	Nicotinamide Adenine Dinucleotide Phosphate
GRSP	Glomalin-Related Soil Protein
